# Schmallenberg Virus in Zoo Ruminants, France and the Netherlands

**DOI:** 10.3201/eid2212.150983

**Published:** 2016-12

**Authors:** Eve Laloy, Cindy Braud, Emmanuel Bréard, Jacques Kaandorp, Aude Bourgeois, Muriel Kohl, Gilles Meyer, Corinne Sailleau, Cyril Viarouge, Stéphan Zientara, Norin Chai

**Affiliations:** Author affiliations: ANSES, Maisons-Alfort, France (E. Laloy, C. Braud, E. Bréard, C. Sailleau, C. Viarouge, S. Zientara);; Safaripark Beekse Bergen, Hilvarenbeek, the Netherlands (J. Kaandorp);; Muséum National d’Histoire Naturelle, Paris, France (A. Bourgeois, M. Kohl, N. Chai);; École Nationale Vétérinaire de Toulouse, Toulouse, France (G. Meyer)

**Keywords:** Orthobunyavirus, wildlife, exotic, zoological park, arbovirus, Schmallenberg virus, SBV, serology, viremia, virus, France, the Netherlands, ruminants, zoonoses

**To the Editor:** Schmallenberg virus (SBV), a new orthobunyavirus of the family *Bunyaviridae*, emerged in August 2011 in northwestern Europe (*1*) and spread to most parts of Europe by *Culicoides* vectors (*2*). Most infections are asymptomatic in adult ruminants, yet fever, milk drop, and diarrhea have been reported (*1*). SBV is responsible for congenital malformations in newborn calves, lambs, and goat kids and has also been associated with abortions and early embryonic losses (*3*). The virus affects domestic livestock, but antibodies to SBV have also been found in free-ranging wild ruminants in several European countries (*3*–*6*) and in wild and exotic ruminants kept in captivity in the United Kingdom and in Austria (*3*–*5*). We carried out a study to investigate the exposure to SBV of wild and exotic ruminants born in Europe and kept in 1 zoological park in France and 1 in the Netherlands.

We tested 42 serum samples (from 39 animals) collected between 2011 and 2014 in the Safaripark Beekse Bergen (SPBB, Hilvarenbeek, the Netherlands) and 18 serum samples (from 15 animals) collected between 2013 and 2015 in the Ménagerie du Jardin des Plantes, Muséum National d’Histoire Naturelle (MJP, Paris, France). First, we determined the presence of SBV-specific antibodies in the samples by ELISA (ELISA ID Screen SBV Competition; ID Vet, Grabels, France) and by virus neutralization test (VNT) according to a protocol previously described (*7*). The 2 methods gave identical results except for 5 samples found negative by ELISA and positive by VNT. Thirty (55.6%) of 54 animals were found to be seropositive by VNT, which is regarded as the standard for SBV detection (Table). Antibodies to SBV were found in 11 (73.3%) of 15 animals from MJP and 19 (48.7%) of 39 animals from SPBB. Positive results were found in samples collected every year during 2011–2015; the earliest positive result was found in a sample collected in September 2011 (SPBB). 

**Table:** Results of virus neutralization testing for Schmallenberg virus among exotic and wild ruminants from 2 zoological parks in France and the Netherlands, 2011–2015*

Common name (species)	No. positive/no. tested	Year(s) of sampling	Animal ages at sampling		Zoological park
			Seropositive	Seronegative	
African buffalo (*Syncerus caffer caffer)*	1/1	2013	3 y		SPBB
Arkal urial sheep (*Ovis aries arkal)*	1/1	2014	5 y		MJP
Axis deer (*Cervus axis)*	0/2	2011–2014		ND, ND	SPBB
Bharal (*Pseudois nayaur)*	2/4	2013, 2014	1 d, 7 y,† 8 y†	10 d, 2 y	MJP
Blackbuck (*Antilope cervicapra*)	0/6	2014		7 mo, 7 y, 15 y, ND, ND, ND	SPBB
Blue wildebeest (*Connochaetes taurinus taurinus)*	3/5	2011	1 y, 6 y, 13 y	1 y, 13 y	SPBB
Common eland (*Taurotragus oryx*)	0/1	2014		1 y	SPBB
Gaur (*Bos gaurus*)	1/1	2015	3 y		MJP
Gemsbok (*Oryx gazella gazella*)	1/1	2011	17 y		SPBB
Markhor *(Capra falconeri*)	2/3	2014	1 y, 10 y	1 y	MJP
Nyala (*Tragelaphus angasii*)	1/2	2012	5 y	ND	SPBB
Père David's deer (*Elaphurus davidianus*)	1/1	2011	15 y		SPBB
Persian fallow deer *(Dama mesopotamica*)	1/1	2013	8 y		SPBB
Pygmy goat (*Capra aegagrus hircus*)	0/1	2014	2 y		MJP
Red forest duiker (*Cephalophus natalensis*)	0/1	2011, 2012		7 y,† 8 y†	SPBB
Rocky mountain goat (*Oreamnus americanus*)	1/1	2014	17 y		MJP
Sable antelope (*Hippotragus niger niger*)	3/3	2011, 2012, 2013	4 y, 5 y,† 6 y,† 7 y,† 10 y		SPBB
Springbok (*Antidorcas marsupialis*)	3/5	2011, 2014	5 y, 14 y, ND	4 y, 5 y	SPBB
Vietnamese sika deer *(Cervus nippon pseudaxis*)	1/1	2014	12 y		SPBB
Vigogna (*Vicugna vicugna)*	1/1	2013	4 y		MJP
Waterbuck (*Kobus ellipsiprymnus ellipsiprymnus*)	1/4	2011, 2014	7 y	6 mo, 4 y, ND	SPBB
Watusi (*Bos taurus taurus watusi*)	0/1	2011		1 y	SPBB
West Caucasian tur (*Capra caucasica caucasica*)	2/2	2014	10 y, 14 y		MJP
Yak (*Bos grunniens grunniens*)	4/5	2012, 2013, 2014	2 y, 3 y, 11 y, ND	1 y	SPBB (4), MJP (1)

*MJP, Ménagerie du Jardin des Plantes (Muséum National d’Histoire Naturelle, Paris, France); ND, not determined; SPBB, Safaripark Beekse Bergen (Hilvarenbeek, the Netherlands). † Animals sampled more than once.

Several seropositive ruminants from MJP were either born in Paris or transferred to Paris from another park in Europe before 2010, which suggests that they were exposed to SBV in Paris. SBV antibodies were found in 3 consecutive samples collected in October 2011, September 2012, and March 2013 from a sable antelope (*Hippotragus niger niger*) in SPBB but also in 3 consecutive samples collected in October 2013, February 2014, and September 2014 in a bharal (*Pseudois nayaur*) from MJP. These data suggest that SBV antibodies can persist for >1 year in these 2 species.

We then performed SBV-specific quantitative reverse transcription PCR targeting the small segment (*8*) of the virus on every sample. One sample from an SBV seronegative blue wildebeest (*Connochaetes taurinus taurinus*) collected in September 2011 in SPBB was positive (quantitation cycle value = 30), whereas the other samples were negative. We also performed several in-house conventional reverse transcription PCR targeting the small, large, and medium segments on the positive sample, which enabled us to retrieve a 2,866-bp partial sequence from the medium segment (deposited in GenBank under accession no. KR828816) and a 1,374-bp partial sequence from the L segment (deposited in GenBank under accession no. KR828815). Genetic analyses based on BLAST (http://blast.ncbi.nlm.nih.gov/Blast.cgi) revealed that the large and medium partial sequences had 100% and 99.79% identity, respectively, with SBV sequences from cows (GenBank accession nos. KM047418 and KP731872, respectively).

Subcutaneous inoculation of serum to adult IFNAR^−/−^ mice, which have been reported to be susceptible to SBV infection (*9*,*10*), did not trigger any clinical sign or seroconversion. No genome could be amplified from their blood.

According to the medical records of SPBB, no clinical signs possibly related to an SBV infection were observed in the ruminants during the period studied. Abortions were reported in MJP in 2 bharals in 2011 and 2012 and in 1 West Caucasian tur (*Capra caucasica caucasica*) in 2013, but no correlation could be drawn between these abortions and the SBV serologic results.

This study demonstrates the circulation of SBV in 18 wild and exotic ruminant species kept in captivity in the Netherlands and in France during 2011–2015. Exposure to the virus may occur even in an urban area (such as central Paris). We report evidence of SBV viremia in a blue wildebeest that was seronegative by ELISA and VNT when the serum was collected. SBV RNA has previously been found in an elk (*6*), but the duration of viremia was not determined. Further investigations are required to determine whether zoo ruminants may play a role in dissemination of SBV.

## References

[1] Hoffmann B, Scheuch M, Höper D, Jungblut R, Holsteg M, Schirrmeier H, et al. Novel orthobunyavirus in cattle, Europe, 2011. Emerg Infect Dis. 2012;18:469–72. 10.3201/eid1803.111905 22376991 PMC3309600

[2] Wernike K, Conraths F, Zanella G, Granzow H, Gache K, Schirrmeier H, et al. Schmallenberg virus-two years of experiences. Prev Vet Med. 2014;116:423–34. 10.1016/j.prevetmed.2014.03.021 24768435

[3] Steinrigl A, Schiefer P, Schleicher C, Peinhopf W, Wodak E, Bagó Z, et al. Rapid spread and association of Schmallenberg virus with ruminant abortions and foetal death in Austria in 2012/2013. Prev Vet Med. 2014;116:350–9. 10.1016/j.prevetmed.2014.03.006 24726407

[4] EFSA (European Food Safety Authority). Schmallenberg virus: state of the art. EFSA journal. 2014;12(5):3681 [cited 2015 May 17]. http://www.efsa.europa.eu/en/efsajournal/pub/3681.htm

[5] Molenaar FM, La Rocca SA, Khatri M, Lopez J, Steinbach F, Dastjerdi A. Exposure of Asian elephants and other exotic ungulates to Schmallenberg virus. PLoS One. 2015;10:e0135532. 10.1371/journal.pone.0135532 26274399 PMC4537289

[6] Larska M, Krzysiak M, Smreczak M, Polak MP, Zmudziński JF. First detection of Schmallenberg virus in elk (*Alces alces*) indicating infection of wildlife in Białowieża National Park in Poland. Vet J. 2013;198:279–81. 10.1016/j.tvjl.2013.08.013 24021421

[7] Bréard E, Lara E, Comtet L, Viarouge C, Doceul V, Desprat A, et al. Validation of a commercially available indirect ELISA using a nucleocapside recombinant protein for detection of Schmallenberg virus antibodies. PLoS One. 2013;8:e53446. 10.1371/journal.pone.0053446 23335964 PMC3546048

[8] Bilk S, Schulze C, Fischer M, Beer M, Hlinak A, Hoffmann B. Organ distribution of Schmallenberg virus RNA in malformed newborns. Vet Microbiol. 2012;159:236–8. 10.1016/j.vetmic.2012.03.035 22516190

[9] Wernike K, Breithaupt A, Keller M, Hoffmann B, Beer M, Eschbaumer M. Schmallenberg virus infection of adult type I interferon receptor knock-out mice. PLoS One. 2012;7:e40380. 10.1371/journal.pone.0040380 22792298 PMC3391294

[10] Ponsart C, Pozzi N, Bréard E, Catinot V, Viard G, Sailleau C, et al. Evidence of excretion of Schmallenberg virus in bull semen. Vet Res (Faisalabad). 2014;45:37. 10.1186/1297-9716-45-37 24708245 PMC3994198

